# Shikonin promotes osteogenic differentiation of periodontal ligament stem cells by modulating the p38 MAPK/ETS transcription factor 1/cementum protein 1 axis in vitro

**DOI:** 10.1002/ccs3.70104

**Published:** 2026-08-08

**Authors:** Zeyu Wu, Chao Shan, Jie Song, Qian Yu, Bing‐jie Lian, Jin Zhao

**Affiliations:** ^1^ Department of Cariology and Endodontics The First Affiliated Hospital of Xinjiang Medical University (The Affiliated Stomatology Hospital of Xinjiang Medical University) Urumqi Xinjiang Province China; ^2^ Xinjiang Uygur Autonomous Region Clinical Research Center for Oral Diseases Urumqi Xinjiang Province China; ^3^ Stomatology Disease Institute of Xinjiang Uyghur Autonomous Region Urumqi Xinjiang Province China

**Keywords:** CEMP1, ELK1, osteogenic differentiation, PDLSCs, Shikonin

## Abstract

Periodontitis is a common disorder that causes tooth loss. Osteogenic differentiation of periodontal ligament stem cells (PDLSCs) is crucial for repairing periodontal defects. This study aimed to investigate the underlying mechanism by which Shikonin promotes the osteogenic differentiation of PDLSCs. PDLSCs were isolated from the periodontal ligaments of orthodontically extracted premolars. Flow cytometry was used to characterize the cell surface markers of PDLSCs. PDLSCs were cultured in osteogenic induction medium and treated with Shikonin. Alkaline phosphatase (ALP) and Alizarin Red S (ARS) staining were used to evaluate ALP activity and assess calcium nodule formation, respectively. Protein levels were determined by Western blot analysis. A dual‐luciferase reporter assay and chromatin immunoprecipitation analysis confirmed the interaction between the ETS transcription factor 1 (ELK1) and *cementum protein 1 (CEMP1)* promoter. Shikonin promoted the osteogenic differentiation of PDLSCs (about 7.6 folds for ALP staining and about 6.9 folds for ARS staining with 1 μm Shikonin, *p* < 0.01). Shikonin‐mediated enhancement of osteogenic differentiation was achieved by promoting the phosphorylation of ELK1 through the activation of the p38 MAPK signaling pathway (*p* < 0.05). Moreover, ELK1 accelerated osteogenic differentiation and transcriptionally activated CEMP1 expression (*p* < 0.05). Activation of the ELK1/CEMP1 axis contributed to Shikonin‐induced osteogenic differentiation of PDLSCs (*p* < 0.05). Shikonin promotes the osteogenic differentiation of PDLSCs in vitro by modulating the p38 MAPK/ELK1/CEMP1 axis.

## Graphical abstract

Our study revealed that Shikonin enhanced osteogenic differentiation by promoting the ELK1/CEMP1 axis via the activation of p38 MAPK signaling in PDLSCs. This indicated that Shikonin may serve as a candidate drug, while the ELK1/CEMP1 axis may be identified as a valuable pathway for promoting the osteogenic differentiation of PDLSCs.

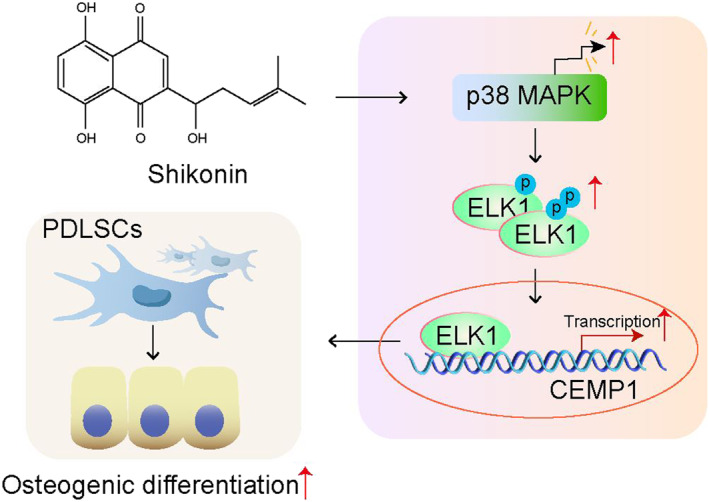

## 1 INTRODUCTION

Periodontitis is a chronic infectious disease that affects oral health. It is triggered by bacterial pathogens and is associated with periodontal tissue destruction and subsequent tooth loss.^1^ Typical symptoms of periodontitis include gingival inflammation (pain and swelling), loss of periodontal attachment, and alveolar bone loss.^2^ Periodontitis has an extremely high prevalence, affecting approximately 50% of the global population, with 11.5% of affected individuals presenting with a severe form of the disease.^3^ Current treatment strategies focus on eliminating local irritants, controlling infection and inflammation, and promoting periodontal tissue healing.^4^ However, periodontal tissue regeneration remains a major challenge to recovery from the disease because of the substantial loss of periodontal bone and the slow rate of bone regeneration during periodontitis, even after drug treatment.^5^ Periodontal ligament stem cells (PDLSCs) are oral mesenchymal stem cells that possess the capacity to differentiate into osteoblasts, cementoblasts, and chondrocytes.^6^ Osteogenic differentiation of PDLSCs plays a critical role in promoting the repair of periodontal bone defects, such as alveolar bone defects, in periodontitis.^7^ However, the mechanisms underlying the osteogenic differentiation of PDLSCs remain elusive and require further investigation to facilitate their further potential clinical application.

Shikonin is the primary bioactive compound isolated from *Lithospermum erythrorhizon*, a traditional Chinese medicinal herb.^8^ Previous studies have shown that Shikonin exerts anti‐inflammatory, antioxidant, and antitumor effects.^9^ Furthermore, Shikonin protects various tissues and organs, including those of the cardiovascular, central nervous, and peripheral nervous systems.^10^ In periodontitis, Shikonin reduces the production of inflammatory cytokines in PDLSCs.^11^ Moreover, Shikonin treatment ameliorates bone resorption and bone loss in a murine model of periodontitis.^12^ Specifically, Shikonin induces the osteogenic differentiation of bone marrow stromal cells and promotes periodontal bone defect repair in rats.^13^ These findings suggest a potential role of Shikonin in the osteogenic differentiation of PDLSCs. However, the precise role and underlying mechanism of Shikonin in the osteogenic differentiation of PDLSCs remains unclear, and elucidating these mechanisms would further expand the understanding of its pharmacological functions.

The ETS transcription factor 1 (ELK1) is a member of the ternary complex factor subgroup within the ETS family of transcription factors.^14^ ELK1 and its downstream targets regulate various biological processes, including cell proliferation and differentiation, particularly in muscle tissues.^15^ ELK1 can be phosphorylated through activation of the p38 MAPK signaling pathway, and subsequently translocates to the nucleus to promote the transcription of its target genes.^16^, ^17^ Aberrant ELK1 expression has been associated with the progression of various diseases, including oral diseases.^18^ In addition, exosomal lncRNA HCP5 derived from human bone marrow mesenchymal stem cells significantly alleviates chronic periodontitis via activation of the p38/ELK1 signaling pathway.^19^ These findings suggest that ELK1 positively regulates the inflammation suppression and osteogenic differentiation of human PDLSCs (hPDLSCs). Moreover, cyclic tensile stress promotes the osteogenic differentiation of hPDLSCs by activating the ERK1/2–ELK1 pathway, thereby enhancing ELK1 expression and phosphorylation.^20^ Shikonin also activates the p38 MAPK/ELK1 pathway in bone marrow stromal cells, thereby inducing osteogenic differentiation and promoting periodontal bone regeneration.^13^ However, whether this signaling pathway mediates the effects of Shikonin on the osteogenic differentiation of PDLSCs remains unknown and warrants further investigation.

Cementum protein 1 (CEMP1) is a key regulatory protein involved in cementogenesis, which supports periodontal tissue development and repair.^21^ CEMP1 promotes osteogenic and cementoblastic differentiation of human gingival fibroblasts.^22^ During periodontitis recovery, a CEMP1‐derived peptide also facilitates periodontal regeneration in a rat model of periodontal defects.^23^ In osteoporotic rats, adipose‐derived mesenchymal stem cells engineered to overexpress CEMP1 exhibit enhanced periodontal regenerative capacity.^24^ However, the direct role of CEMP1 in the osteogenic differentiation of PDLSCs remains elusive, and requires further investigation. Similarly, whether Shikonin regulates CEMP1 expression in PDLSCs remains unclear. Additionally, bioinformatic analysis using the JASPAR database predicted that ELK1 may bind to the *CEMP1* promoter, and the interaction between ELK1 and CEMP1 during osteogenic differentiation warrants further investigation.

Therefore, this study aimed to clarify the specific role of Shikonin in the osteogenic differentiation of PDLSCs. We hypothesized that Shikonin promotes osteogenic differentiation by enhancing the ELK1/CEMP1 axis through activation of the p38 MAPK signaling pathway in PDLSCs. Exploring the role and underlying mechanism of Shikonin in osteogenic differentiation may help in identifying potential therapeutic targets and contribute to the development of novel treatment strategies for periodontal regeneration.

## 2 MATERIAL AND METHODS

### 2.1 Extraction and isolation of PDLSCs

PDLSCs were isolated from the periodontal ligaments of orthodontically extracted premolars obtained from healthy donors undergoing orthodontic treatment, following approval from the Ethics Committee of the First Affiliated Hospital of Xinjiang Medical University (Ethics Approval No. K202304‐19). All donors were informed of the study procedures and provided written informed consent before sample collection. Briefly, periodontal ligaments were scraped from the middle third of the tooth roots, cut into approximately 2 mm^3^ fragments, and digested with collagenase type I (#A417694, 3 mg/mL, Sangon Biotech, Shanghai, China) and dispase (#A002100, 4 mg/mL, Sangon Biotech) for 15 min at 37°C to isolate PDLSCs, as previously described.^25^, ^26^

### 2.2 Cell culture and treatment

HEK293T cells were purchased from ATCC (Cat# CRL‐3216, RRID: CVCL_0063) in 2023, and PDLSCs were maintained in alpha minimum essential medium (Gibco, Waltham, MA, USA) supplemented with 10% fetal bovine serum (Gibco) and 1% penicillin–streptomycin in a CO_2_ incubator at 37°C. The cells were confirmed to be free of *mycoplasma* contamination and were authenticated by short tandem repeat profiling. Third‐passage PDLSCs were used for subsequent experiments. PDLSCs (2 × 10^4^ cells/well) were seeded into 6‐well plates and cultured to approximately 80% confluence before the induction of osteogenic or adipogenic differentiation. The culture medium was then replaced with osteogenic induction medium (OM; Cyagen) for 14 or 28 days or adipogenic induction medium (AM; Cyagen) for 14 days. Subsequently, according to previous studies,^26^, ^27^ cells were fixed with 4% paraformaldehyde and stained with alkaline phosphatase (ALP), Alizarin Red S (ARS), or Oil Red O to evaluate mineralized nodule formation or lipid droplet accumulation. To investigate the effects of Shikonin (#S115193, Aladdin Biotech) on the osteogenic differentiation of PDLSCs, Shikonin was added to the OM at increasing concentrations (0, 0.25, 0.5, 1, and 2 μM) during osteogenic induction, according to a previously reported protocol.^12^ Furthermore, to inhibit the p38 MAPK signaling pathway, PDLSCs were treated with the p38 MAPK inhibitor adezmapimod (SB 203580, 10 μM; #HY‐10256, MCE) for 48 h in OM.

### 2.3 Cell viability assay

A Cell Counting Kit‐8 (CCK‐8) (#CK04, Solarbio Biotech) was used to assess cell viability. PDLSCs (1 × 10^4^ cells/well) were seeded into 96‐well plates and incubated for 24 h at 37°C. Following the designated treatments, 10 μL of CCK‐8 solution was added to each well, and the cells were incubated for 2 h. The optical density at 450 nm was subsequently measured using a microplate reader (ReadMax 1900; Shanpu Biotech).

### 2.4 Flow cytometry analysis

Flow cytometry was used to characterize the cell surface marker expression of PDLSCs, including the positive markers CD90, CD105, and CD29, and the negative hematopoietic markers CD31 and CD45, as previously described.^28^ Briefly, PDLSCs (5 × 10^5^ cells) were incubated with anti‐CD90 (#202526, 1:100, BioLegend), anti‐CD105 (#323206, 1:100, BioLegend), anti‐CD29 (#303008, 1:100, BioLegend), anti‐CD31 (#303106, 1:100, BioLegend), and anti‐CD45 (#368511, 1:100, BioLegend) antibodies for 1 h at 4°C in the dark. Apoptosis was evaluated using an Annexin V‐FITC Apoptosis Detection Kit (#CA1020, Solarbio Biotech). PDLSCs (5 × 10^5^ cells) were double‐stained with FITC‐conjugated Annexin V (5 μL) and propidium iodide (PI; 5 μL) for 15 min in the dark. Cells that were FITC Annexin V‐positive and PI‐negative were classified as early apoptotic, whereas those that were FITC Annexin V‐positive and PI‐positive were classified as late apoptotic. Flow cytometry was performed using an Epics XL cytometer (Beckman Coulter).

### 2.5 Cell transfection

To silence ELK1 and CEMP1, lentiviral vectors (pLKO.1‐puro) containing shRNA targeting ELK1 (sh‐ELK1, 5′‐GCAACATAAGTTCGTCACC‐3′) and CEMP1 (sh‐CEMP1, 5′‐GGTGGAGGTGGTCAGCTTT‐3′), or a scrambled negative control (sh‐NC, 5′‐TTCTCCGAACGTGTCACGT‐3′), were obtained from GenePharma. To overexpress ELK1 (oe‐ELK1) or CEMP1 (oe‐CEMP1), the full‐length coding sequences of ELK1 (NM_001114123.3) and CEMP1 (NM_001048212.3) were synthesized and cloned into the pCDH‐CMV‐MCS‐EF1‐Puro vector (GenePharma). An empty vector served as the negative control (oe‐NC). PDLSCs were seeded into 6‐well plates (8 × 10^5^ cells/well) and cultured to approximately 70% confluence before being infected with the corresponding lentiviral vectors in the presence of 4 μg/mL polybrene at a multiplicity of infection of 20.

### 2.6 ALP and ARS staining

After osteogenic induction, PDLSCs were fixed with 4% paraformaldehyde for 20 min and stained with a BCIP/NBT ALP color development kit (#C3206, Beyotime Biotech) for 30 min at 37°C. For ARS staining, after fixation with 4% paraformaldehyde, PDLSCs were stained with ARS solution (#C0148S, Beyotime Biotech) for 30 min at 37°C. Subsequently, ALP staining and extracellular matrix mineralized nodule formation were observed and imaged using an optical microscope (CX43, Olympus, Tokyo, Japan). The staining intensity and positive staining area were quantitatively analyzed using the ImageJ software (NIH). For each group, at least three fields were randomly selected for analysis.

### 2.7 Oil red O staining

To evaluate lipid droplet formation, AM‐induced PDLSCs were fixed with 4% paraformaldehyde for 20 min and then stained with the Oil Red O solution from an Oil Red O Stain Kit (#G1262, Solarbio Biotech). Lipid droplet formation was observed and photographed under a microscope (Olympus), and the positive staining area was quantitatively analyzed using ImageJ software (NIH). At least three random fields were analyzed for each group.

### 2.8 Live/dead staining assay

The Calcein‐AM/PI Live/Dead Cell Double Stain Kit (#CA1630; Solarbio Biotech) was used to evaluate cell viability. Briefly, PDLSCs (2 × 10^4^ cells) were incubated with a working solution containing Calcein‐AM (1 μM) and PI (2 μg/mL) for 30 min in the dark. Fluorescence images were captured using a confocal laser‐scanning microscope (FV5000, Olympus). The fluorescence images were quantitatively analyzed using the ImageJ software (NIH). A minimum of three random fields were analyzed for each group.

### 2.9 Western blot analysis

Cells were lysed on ice in Thermo Scientific RIPA buffer (Cat# 89900) supplemented with protease inhibitors for 30 min at 4°C, and protein concentrations were determined using a BCA Protein Quantification Kit (#20201ES76, Yeasen Biotech). Equal amounts of protein (30 μg per sample) were separated by sodium dodecyl sulfate‐polyacrylamide gel electrophoresis and transferred onto polyvinylidene difluoride membranes. The membranes were then incubated overnight at 4°C with primary antibodies, followed by incubation with secondary antibodies for 1 h at room temperature (25°C). The chemiluminescent signals were detected using Super ECL Western Blotting Substrate (#HXP1868, Huaxingbio Biotech). The primary antibodies included anti‐Runx2 (Runx family transcription factor 2; 1:1000, #ab236639), anti‐Osterix (Osx; 1:1000, #ab209484), anti‐ALP (1:1000, #ab307726), anti‐collagen type I (Col1; 1:1000, #ab138492), anti‐phosphorylated p38 (p‐p38; 1:1000, #ab4822), anti‐p38 (1:1000, #ab178867), anti‐phosphorylated ELK1 (p‐ELK1; 1:1000, #ab218133), anti‐ELK1 (1:1000, #ab125085), anti‐ERK (1:1000, #ab184699), anti‐phosphorylated ERK (p‐ERK; 1:1000, #ab76299), anti‐CEMP1 (1:1000, #ab169514), and anti‐β‐actin (1:1000, #ab8226), all purchased from Abcam (Cambridge, UK). β‐actin served as the loading control.

### 2.10 Dual‐luciferase reporter assay

To confirm the binding of ELK1 to the *CEMP1* promoter, the predicted *CEMP1* promoter fragment containing the ELK1‐binding site upstream of the transcription start site, along with its corresponding mutant sequence, was amplified and cloned into the pGL3 vector (CEMP1‐WT or CEMP1‐MUT; Promega, Madison, WI, USA). HEK293T cells were co‐transfected with the *CEMP1* promoter reporter plasmid alongside either the oe‐ELK1 or oe‐NC vectors. After 48 h, the luciferase activity was measured using a Dual‐Luciferase Reporter Assay System (Promega).

### 2.11 Chromatin immunoprecipitation (ChIP) assay

The interaction between ELK1 and the *CEMP1* promoter was verified using a ChIP Assay Kit (#P2078, Beyotime Biotech) according to the manufacturer's instructions. A total of 1 × 10^6^ PDLSCs per dish were crosslinked with 1% formaldehyde for 15 min and quenched with glycine for 5 min. Subsequently, the cells were lysed, sonicated, and enzymatically digested to obtain DNA fragments ranging from 200 to 1000 bp according to the manufacturer's instructions. The processed samples were then incubated with either anti‐ELK1 (#27420‐1‐AP, proteintech) or anti‐immunoglobulin G (IgG) (#ab172730, Abcam) antibodies for 12 h at 4°C. After elution and purification, quantitative PCR was performed to confirm the ELK1‐binding sites.

### 2.12 Statistical analysis

Statistical analyses were performed using SPSS (version 20.0; SPSS Inc.). Tests for normality and homogeneity of variance were performed before conducting the statistical analyses. Student's *t*‐test was used to compare two groups, whereas one‐way analysis of variance followed by Tukey's post hoc test was used for comparisons among multiple groups. Data are presented as the mean ± standard deviation. Each experiment was independently performed at least thrice. *N* = 3 indicates three independent biological replicates, each consisting of three technical replicates. Differences were considered statistically significant when *p* < 0.05.

## 3 RESULTS

### 3.1 Characterization and identification of PDLSCs

**FIGURE 1:**
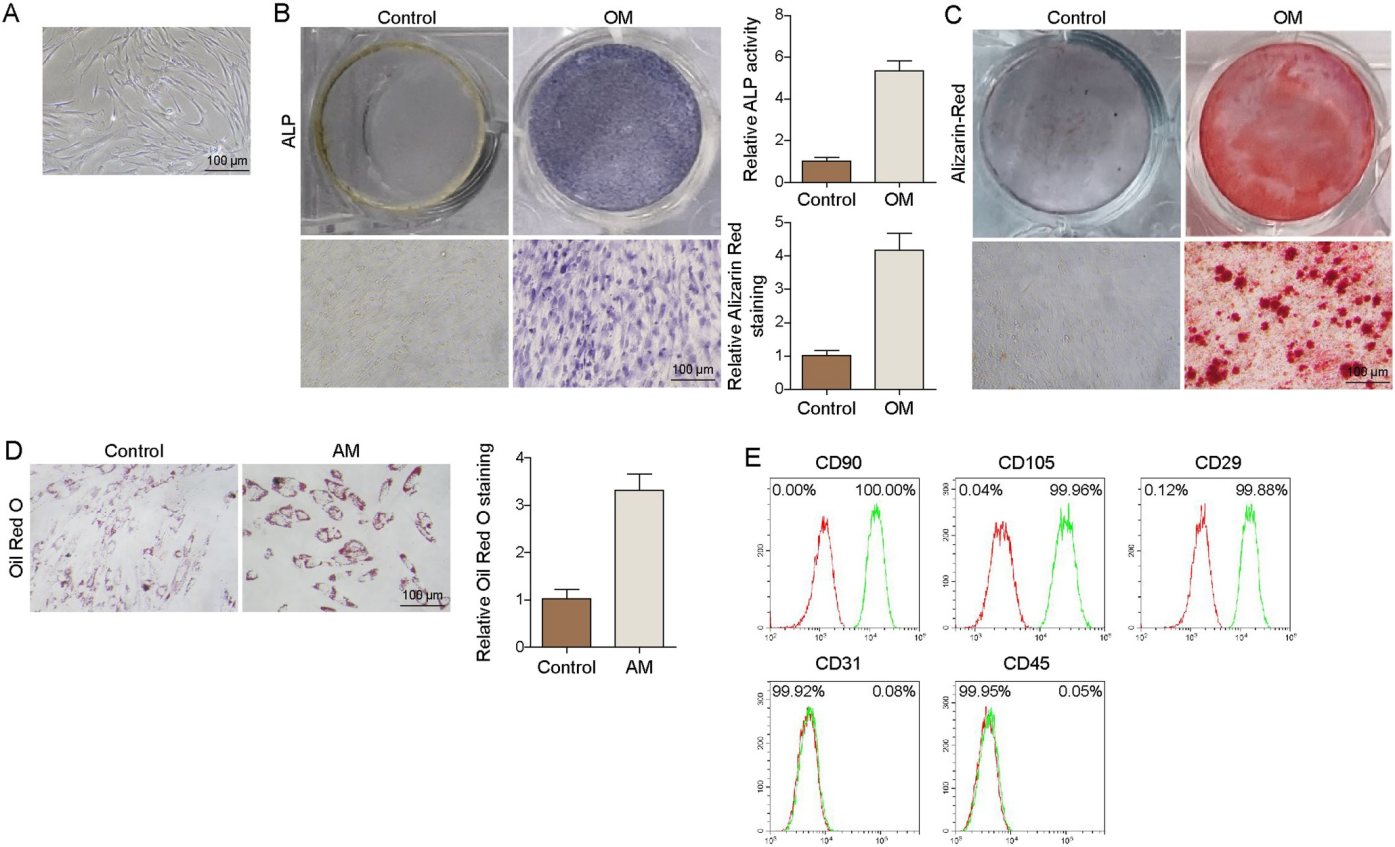
Characterization and identification of PDLSCs. PDLSCs were isolated from the periodontal ligaments of orthodontically extracted premolars obtained from healthy donors. (A) PDLSC morphology was observed using an optical microscope (scale bar = 100 μm). PDLSCs were cultured in osteogenic induction medium (OM) or adipogenic induction medium (AM) for 14 or 28 days (panels B–E). (B) ALP staining was performed to evaluate ALP activity (scale bar = 100 μm). (C) Mineralized nodule formation was evaluated by Alizarin Red S staining (scale bar = 100 μm). (D) Oil red O staining was performed to evaluate lipid droplet formation (scale bar = 100 μm). (E) The expression of the mesenchymal stem cell markers CD90, CD105, and CD29, and the hematopoietic cell markers CD31 and CD45 was analyzed by flow cytometry. Tests for normality and homogeneity of variance were performed before statistical analysis. Student's *t*‐test was used to compare two groups. Data are presented as the mean ± standard deviation. Each experiment was independently performed at least thrice. *n* = 3 indicates three independent biological replicates, each consisting of three technical replicates. Statistical significance is indicated as follows: ***p < 0.001. ALP, Alkaline phosphatase.

PDLSCs were isolated from the periodontal ligaments of orthodontically extracted donor premolars to investigate the underlying mechanisms of osteogenic differentiation. Microscopy showed that PDLSCs exhibited adherent growth with a typical spindle‐shaped morphology (Figure 1A). PDLSCs were then cultured in OM or AM for 14 or 28 days to induce osteogenic or adipogenic differentiation, respectively. As shown in Figure 1B,C, compared to the control group, PDLSCs cultured in OM exhibited significantly increased ALP activity and mineralized nodule formation, indicating successful osteogenic differentiation. In addition, Oil Red O staining showed that PDLSCs cultured in AM accumulated significantly more lipid droplets than the control group, demonstrating the adipogenic differentiation potential of PDLSCs (Figure 1D). Furthermore, the isolated PDLSCs were positive for CD90, CD105, and CD29, but negative for CD31 and CD45 (Figure 1E). Collectively, these findings confirmed the mesenchymal characteristics and purity of the isolated PDLSCs.

### 3.2 Shikonin promoted osteogenic differentiation of PDLSCs

**FIGURE 2:**
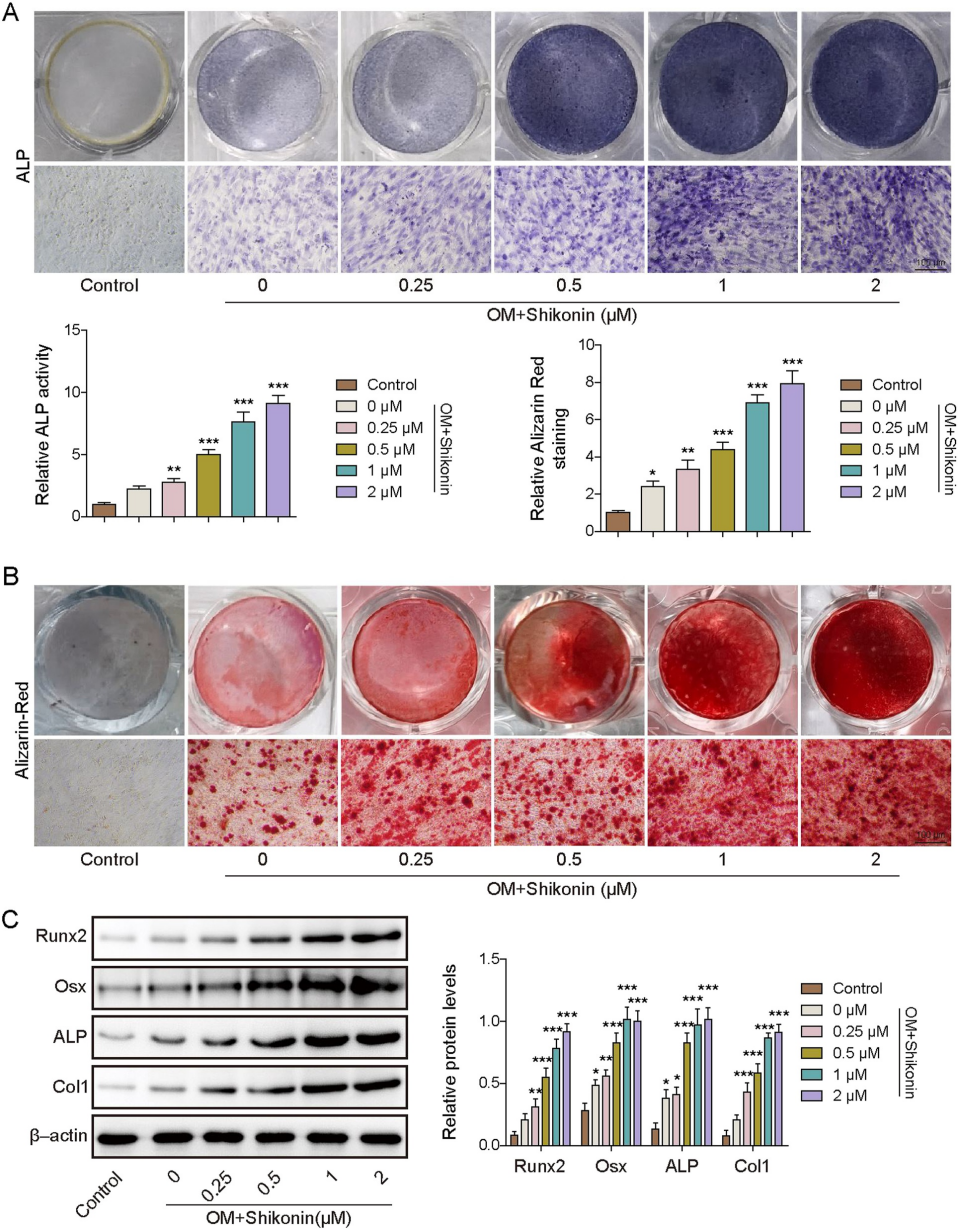
Shikonin promoted osteogenic differentiation of PDLSCs. PDLSCs were treated with Shikonin at concentrations of 0, 0.25, 0.5, 1, and 2 μM during OM induction. (A) ALP staining was performed to evaluate ALP activity (scale bar = 100 μm). (B) Mineralized nodule formation was evaluated by Alizarin Red S staining (scale bar = 100 μm). (C) The protein expression levels of the osteogenic markers Runx2, Osx, ALP, and Col1 were determined by Western blot analysis. Tests for normality and homogeneity of variance were performed before statistical analysis. One‐way ANOVA followed by Tukey's post hoc test was used for comparisons among multiple groups. Data are presented as the mean ± standard deviation. Each experiment was independently performed at least thrice. *n* = 3 indicates three independent biological replicates, each consisting of three technical replicates. Statistical significance is indicated as follows: *p < 0.05, **p < 0.01, and ***p < 0.001. ALP, Alkaline phosphatase.

To investigate the effects of Shikonin on the osteogenic differentiation of PDLSCs, the cells were treated with various concentrations of Shikonin (0, 0.25, 0.5, 1, and 2 μM) in OM. Shikonin treatment dose‐dependently increased ALP activity and enhanced mineralized nodule formation in PDLSCs cultured in OM (Figure 2A,B). Furthermore, compared with the control group, the protein expression levels of the osteogenic markers Runx2, Osx, ALP, and Col1 were increased in OM‐cultured PDLSCs, and co‐treatment with Shikonin further enhanced their expression in a concentration‐dependent manner (Figure 2C). Collectively, these results indicate that Shikonin significantly promotes the osteogenic differentiation of PDLSCs in a dose‐dependent manner.

### 3.3 Shikonin enhanced osteogenic differentiation by promoting ELK1 phosphorylation via activation of the p38 MAPK pathway in PDLSCs

**FIGURE 3:**
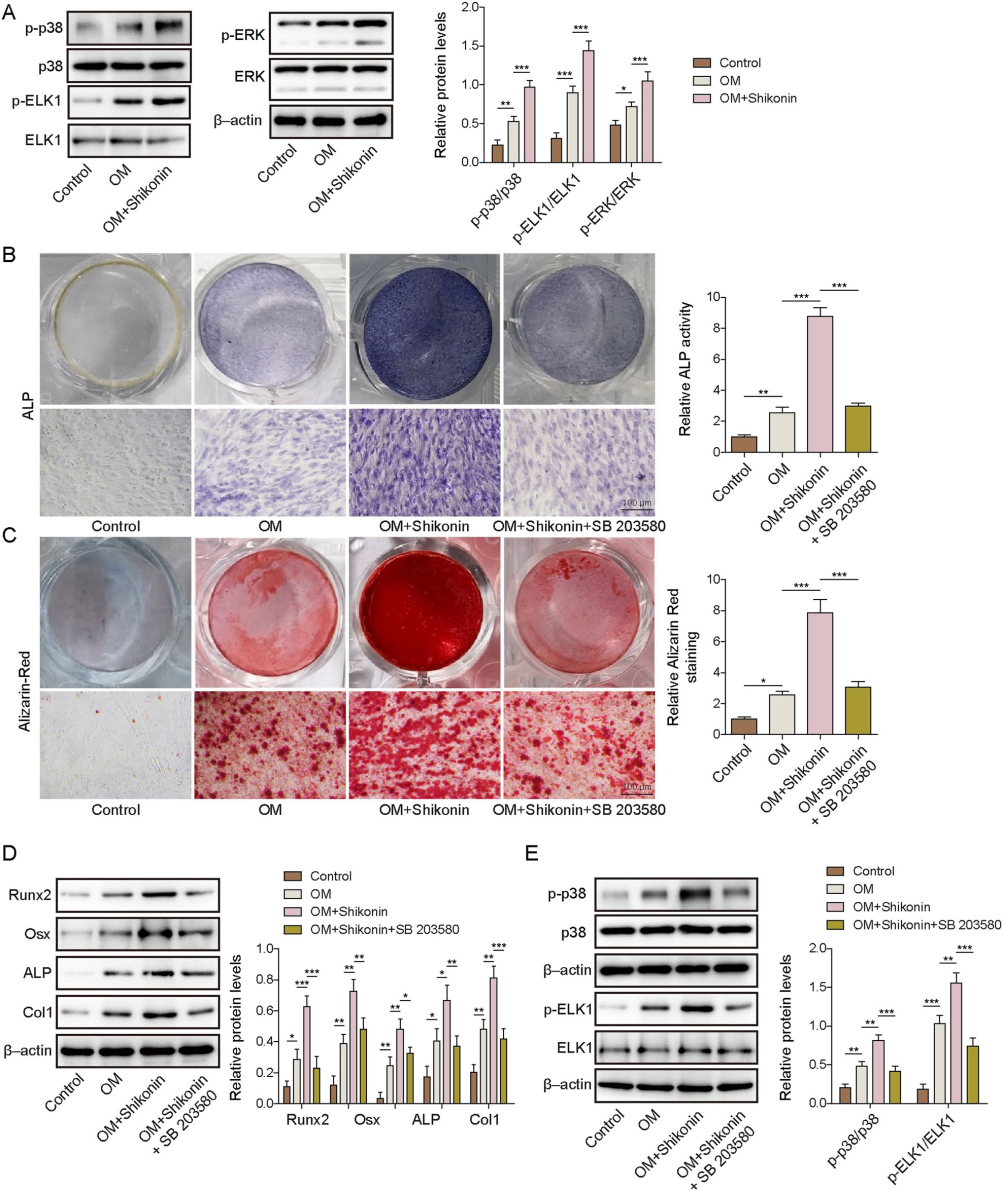
Shikonin enhanced osteogenic differentiation by promoting ELK1 phosphorylation via activation of the p38 MAPK pathway in PDLSCs. PDLSCs were treated with Shikonin (1 μM) during OM induction. (A) The protein expression levels of p38, p‐p38, ELK1, p‐ELK1, ERK, and p‐ERK were determined by Western blot analysis. PDLSCs were treated with the p38 MAPK inhibitor adezmapimod (SB 203580, 10 μM) under Shikonin treatment in OM (panels B–E). (B) ALP staining was performed to evaluate ALP activity (scale bar = 100 μm). (C) Mineralized nodule formation was evaluated by Alizarin Red S staining (scale bar = 100 μm). (D) The protein expression levels of the osteogenic markers Runx2, Osx, ALP, and Col1 were determined by Western blot analysis. (E) The protein expression levels of p38, p‐p38, ELK1, and p‐ELK1, were determined by Western blot analysis. Normality distribution tests and homogeneity of variance tests were conducted before the analysis. One‐way ANOVA followed by Tukey's post hoc test was used for comparisons among multiple groups. Data are presented as the mean ± standard deviation. Each experiment was independently performed at least thrice. *n* = 3 indicates three independent biological replicates, each consisting of three technical replicates. Statistical significance is indicated as follows: *p < 0.05, **p < 0.01, and ***p < 0.001. ALP, Alkaline phosphatase; p‐p38, phosphorylation of p38.

At concentrations ranging from 0 to 2 μM, Shikonin did not affect cell viability or apoptosis (Supporting Information S1 Figure S1A–C). However, at a concentration of 10 μM, Shikonin decreased cell viability and slightly increased apoptosis (Supporting Information S1 Figure S1A–C). These findings indicate that Shikonin was not cytotoxic at concentrations below 2 μM, whereas 10 μM exhibited measurable cytotoxicity. Because no significant difference in osteogenic differentiation was observed between the 1 and 2 μM Shikonin groups, whereas 10 μM of Shikonin exhibited cytotoxic effects, 1 μM was selected for subsequent experiments to achieve maximal efficacy at the lowest effective concentration. Therefore, in this section, PDLSCs were divided into the control, OM, and OM + Shikonin (1 μM) groups. As shown in Figure 3A, OM induction markedly increased the phosphorylation of p38 (p‐p38), ELK1 (p‐ELK1), and ERK (p‐ERK), and Shikonin supplementation further increased the p‐p38/p38, p‐ELK1/ELK1, and p‐ERK/ERK ratios. To further investigate the role of p38 MAPK signaling pathway in the Shikonin‐mediated promotion of osteogenic differentiation in PDLSCs, the cells were treated with the p38 MAPK inhibitor SB 203580 and Shikonin in OM. Inhibition of the p38 MAPK pathway reversed the Shikonin‐induced increases in ALP activity and mineralized nodule formation in OM‐treated PDLSCs (Figure 3B,C). Consistently, the Shikonin‐induced upregulation of the osteogenic markers Runx2, Osx, ALP, and Col1 was abolished by the inhibition of the p38 MAPK pathway in PDLSCs cultured in OM (Figure 3D). Compared with the OM + Shikonin group, p38 inhibition significantly decreased expression levels of p‐p38/p38 and p‐ELK1/ELK1 in Shikonin‐treated PDLSCs (Figure 3E). Collectively, these findings indicate that Shikonin promotes the osteogenic differentiation of PDLSCs by enhancing ELK1 phosphorylation through activation of the p38 MAPK pathway.

### 3.4 ELK1 facilitated osteogenic differentiation and transcriptionally activated CEMP1 in PDLSCs

**FIGURE 4:**
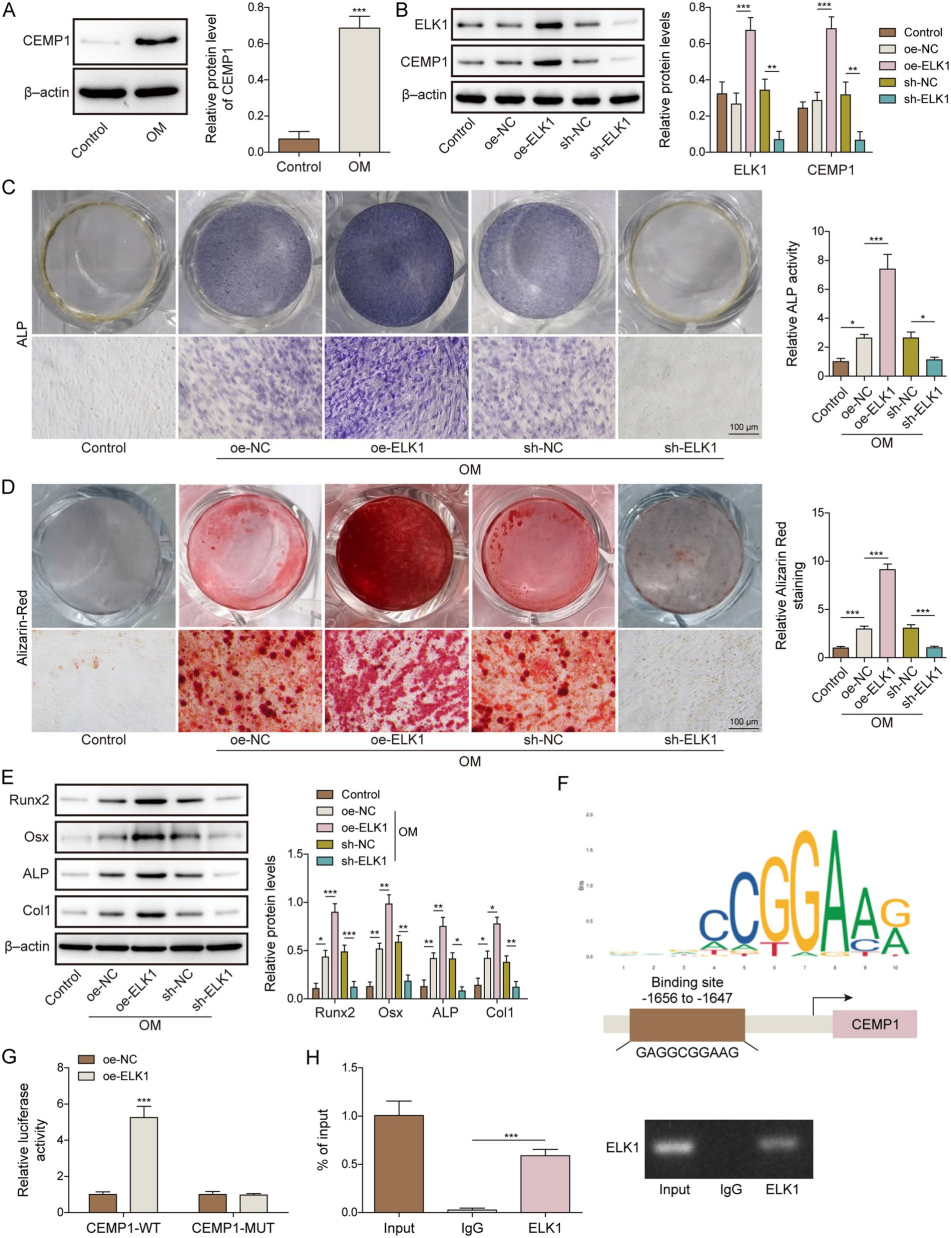
ELK1 facilitated osteogenic differentiation and transcriptionally activated CEMP1 in PDLSCs. PDLSCs were cultured in OM. (A) CEMP1 protein expression was determined by Western blot analysis. PDLSCs were transduced with lentiviral vectors carrying oe‐ELK1, sh‐ELK1, or the corresponding negative control vectors under OM conditions (panels B–E). (B) The protein expression levels of ELK1 and CEMP1 were determined by Western blot analysis. (C) ALP staining was performed to evaluate ALP activity (scale bar = 100 μm). (D) Mineralized nodule formation was evaluated by Alizarin Red S staining (scale bar = 100 μm). (E) The protein expression levels of the osteogenic markers Runx2, Osx, ALP, and Col1 were determined by Western blot analysis. (F) The JASPAR database predicted putative ELK1‐binding sites within the *CEMP1* promoter. (G) A dual‐luciferase reporter assay was performed to validate the interaction between ELK1 and the *CEMP1* promoter. (H) Interaction between ELK1 and the *CEMP1* promoter was further confirmed by chromatin immunoprecipitation (ChIP) assay, and the enrichment of the *CEMP1* promoter is presented as % input. Tests for normality and homogeneity of variance were performed before statistical analysis. Student's *t*‐test was used to compare two groups, whereas one‐way ANOVA followed by Tukey's post hoc test was used for comparisons among multiple groups. Data are presented as the mean ± standard deviation. Each experiment was independently performed at least thrice. *n* = 3 indicates three independent biological replicates, each consisting of three technical replicates. Statistical significance is indicated as follows: *p < 0.05, **p < 0.01, and ***p < 0.001. ALP, Alkaline phosphatase; CEMP1, cementum protein 1.

As shown in Figure 4A, PDLSCs cultured in OM exhibited significantly higher CEMP1 protein expression than the control group. PDLSCs were then subjected to ELK1 overexpression or silencing using lentiviral vectors under OM conditions. ELK1 overexpression markedly increased the protein expression levels of ELK1 and CEMP1, whereas ELK1 silencing significantly decreased their expression (Figure 4B). Furthermore, ELK1 overexpression increased ALP activity and mineralized nodule formation in PDLSCs cultured in OM, whereas ELK1 silencing attenuated these effects (Figure 4C,D). Similarly, ELK1 overexpression increased the protein expression levels of the osteogenic markers Runx2, Osx, ALP, and Col1, whereas ELK1 silencing reduced their expression in OM‐induced PDLSCs (Figure 4E). In addition, as shown in Figure 4F, the JASPAR database (http://jaspar.genereg.net/) predicted putative ELK1‐binding sites within the *CEMP1* promoter. The dual‐luciferase reporter assay further confirmed the interaction between ELK1 and the *CEMP1* promoter, as luciferase activity was significantly increased by oe‐ELK1 transfection in the CEMP1‐WT group but not in the CEMP1‐MUT group (Figure 4G). Furthermore, the ChIP assay verified this interaction. Specifically, enrichment of the *CEMP1* promoter (% input) was significantly higher in the anti‐ELK1 antibody group than in the anti‐IgG antibody group (Figure 4H). Moreover, a PDLSC model with stable CEMP1 knockdown was established using lentiviral transduction, followed by treatment with OM and Shikonin. Under OM treatment, CEMP1 expression increased, accompanied by increased expression of the osteogenic markers Runx2, Osx, ALP, and Col1 (Supporting Information S1 Figure S2A–D). Shikonin treatment further enhanced these changes, whereas CEMP1 knockdown reversed these effects (Supporting Information S1 Figure S2A–D). These findings indicate that Shikonin promotes the osteogenic differentiation of PDLSCs, at least in part, by regulating CEMP1 expression. Collectively, these results demonstrate that ELK1 positively regulates osteogenic differentiation and transcriptionally activates CEMP1 transcription in PDLSCs.

### 3.5 The ELK1/CEMP1 axis was implicated in the Shikonin‐enhanced osteogenic differentiation of PDLSCs

**FIGURE 5:**
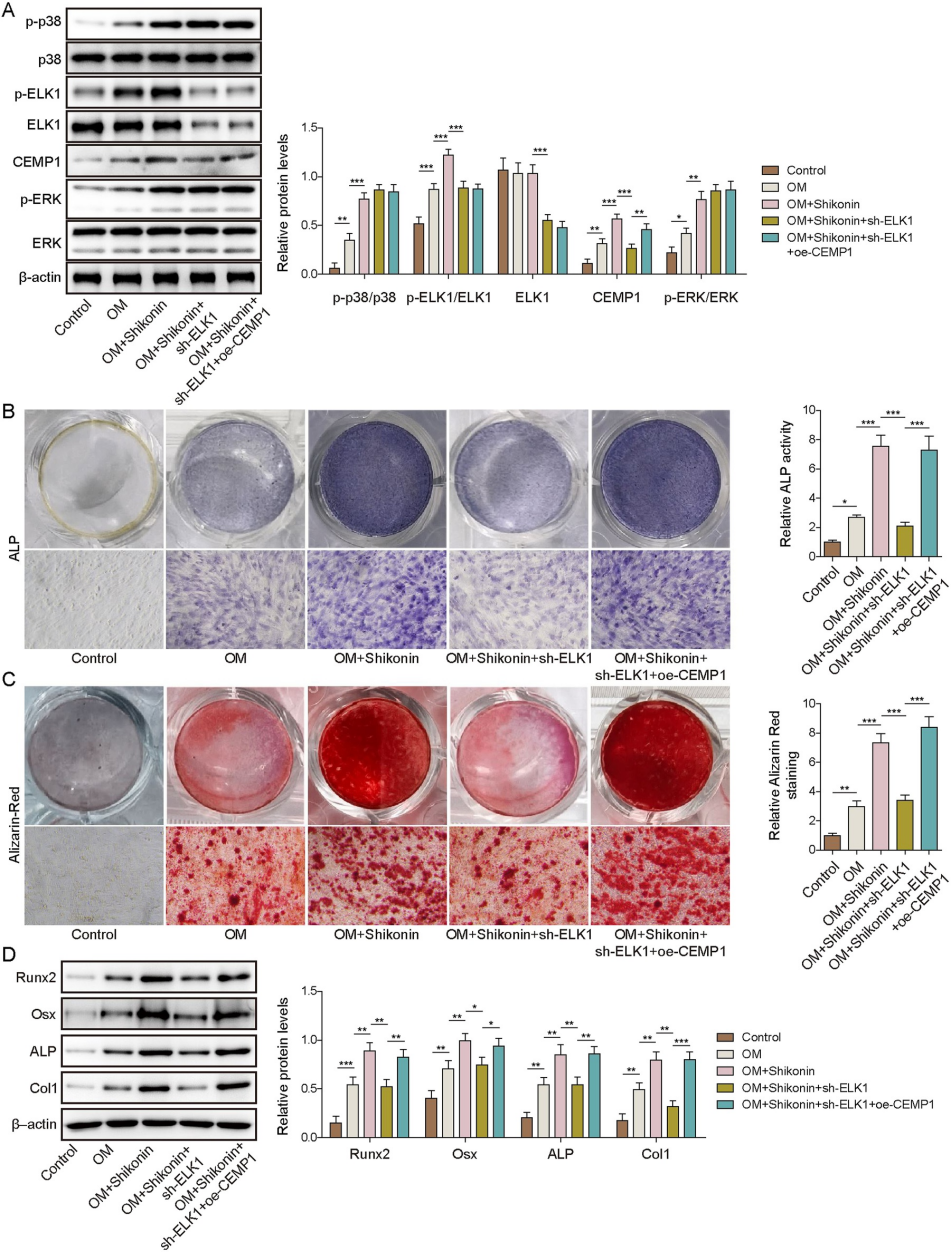
The ELK1/CEMP1 axis was implicated in Shikonin‐enhanced osteogenic differentiation of PDLSCs. PDLSCs were transduced with lentiviral vectors carrying sh‐ELK1 alone or in combination with oe‐CEMP1 under Shikonin treatment in OM. The cells were divided into the control, OM, OM + Shikonin, OM + Shikonin + sh‐ELK1, and OM + Shikonin + sh‐ELK1 + oe‐CEMP1 groups. (A) The protein expression levels of p38, phosphorylation of p38, ELK1, p‐ELK1, CEMP1, ERK, and p‐ERK were determined by Western blot analysis. (B) ALP staining was performed to evaluate ALP activity (scale bar = 100 μm). (C) Mineralized nodule formation was evaluated by Alizarin Red S staining (scale bar = 100 μm). (D) The protein expression levels of the osteogenic markers Runx2, Osx, ALP, and Col1 were determined by Western blot analysis. Tests for normality and homogeneity of variance were performed before statistical analysis. One‐way ANOVA followed by Tukey's post hoc test was used for comparisons among multiple groups. Data are presented as the mean ± standard deviation. Each experiment was independently performed at least thrice. *n* = 3 indicates three independent biological replicates, each consisting of three technical replicates. Statistical significance is indicated as follows: *p < 0.05, **p < 0.01, and ***p < 0.001. ALP, Alkaline phosphatase; CEMP1, cementum protein 1.

To investigate the interaction between Shikonin and the ELK1/CEMP1 axis during osteogenic differentiation of PDLSCs, the cells were transduced with lentiviral vectors carrying sh‐ELK1 and oe‐CEMP1 under OM and Shikonin treatment. The experimental groups included the control, OM, OM + Shikonin, OM + Shikonin + sh‐ELK1, and OM + Shikonin + sh‐ELK1 + oe‐CEMP1 groups. Compared with the OM + Shikonin group, ELK1 silencing significantly decreased the protein expression levels of ELK1 and CEMP1, as well as the p‐ELK1/ELK1 ratio, but did not affect the expression of p‐p38/p38 and p‐ERK/ERK. Co‐expression of CEMP1 restored only CEMP1 expression and did not affect the changes in ELK1 phosphorylation or the upstream signaling proteins in Shikonin‐treated PDLSCs cultured in OM (Figure 5A). Compared with the OM + Shikonin group, ELK1 silencing decreased ALP activity and mineralized nodule formation, whereas CEMP1 overexpression partially rescued these inhibitory effects (Figure 5B,C). Moreover, ELK1 silencing significantly reduced the protein expression levels of the osteogenic markers Runx2, Osx, ALP, and Col1 compared with the OM + Shikonin group, whereas additional CEMP1 overexpression partially restored their expression (Figure 5D). Collectively, these findings indicate that CEMP1 functions as a downstream effector of ELK1 during Shikonin‐induced osteogenic differentiation of PDLSCs. Because CEMP1 overexpression partially rescued the inhibitory effects of ELK1 silencing, additional downstream mechanisms may also contribute to the osteogenic effects mediated by ELK1.

## 4 DISCUSSION

Periodontitis is one of the most common oral diseases and represents a substantial global public health burden, affecting approximately 50% of the world's population.^29^ Periodontitis is a chronic inflammatory disease characterized by the progressive destruction of periodontal tissues, including the gingiva, periodontal ligaments, and alveolar bone.^30^ The primary goal of periodontitis management is to restore the structure and function of periodontal tissues, in addition to controlling the infection.^31^ As progenitor cells, PDLSCs play a vital role in orchestrating periodontal tissue regeneration during recovery from periodontitis.^6^ Osteogenic differentiation of PDLSCs is considered a key determinant of alveolar bone regeneration and periodontal bone defect repair, and enhancing this process may accelerate periodontal tissue regeneration.^32^ In the present study, we isolated hPDLSCs and demonstrated that Shikonin promoted their osteogenic differentiation, potentially through regulation of the p38 MAPK/ELK1/CEMP1 axis. These findings expand the understanding of the pharmacological effects and underlying mechanisms of Shikonin in promoting osteogenic differentiation. Moreover, the ELK1/CEMP1 axis may represent a potential molecular target enhancing the osteogenic differentiation of PDLSCs.

Shikonin is the principal bioactive compound isolated from the roots of *L*. *erythrorhizon*, a traditional Chinese medicinal herb with wound‐healing properties.^33^ Shikonin has been shown to exert anti‐inflammatory, antioxidant, and antitumor effects.^9^ In periodontitis, Shikonin has been reported to suppress inflammatory responses and promote osteogenic differentiation in bone marrow stromal cells.^11^, ^13^ In the present study, we demonstrated that Shikonin promoted osteogenic differentiation and upregulated the expression of the osteogenic markers, Runx2, Osx, ALP, and Col1, in PDLSCs cultured in OM. These findings are consistent with previous reports in MC3T3‐E1 cells and bone marrow stromal cells.^13^, ^34^ Furthermore, regarding the potential clinical translation of Shikonin for periodontal therapy and other tissue regeneration applications, it is important to address translational challenges, including limited bioavailability, environmental instability, and appropriate drug delivery strategies. Potential approaches include β‐1,3‐1,6‐glucan‐based formulations and local drug delivery systems, such as injectable drug‐loaded hydrogels administered into periodontal pockets.^35^, ^36^ Collectively, this study demonstrates that Shikonin promotes the osteogenic differentiation of PDLSCs, highlighting a previously unrecognized pharmacological function.

ELK1 belongs to the ETS family of transcription factors and plays a critical role in regulating the proliferation and differentiation of bone‐derived cells.^37^ ELK1 phosphorylation is promoted through activation of the p38 MAPK signaling pathway, thereby facilitating the transcription of target genes involved in the proliferation and differentiation of bone marrow mesenchymal stem cells.^38^, ^39^ In hPDLSCs, activation of the p38 MAPK signaling pathway and increased ELK1 phosphorylation have been reported during enhanced osteogenic differentiation.^19^ Consistent with these findings, our study demonstrated activation of the p38 MAPK signaling pathway and increased p‐ELK1/ELK expression during induction of osteogenic differentiation of PDLSCs. Moreover, Shikonin has been reported to activate the p38 MAPK/ELK1 axis in bone marrow stromal cells, thereby promoting osteogenic differentiation.^13^ Similarly, results showed that Shikonin enhanced the osteogenic differentiation of PDLSCs through activation of the p38 MAPK/ELK1 pathway, whereas pharmacological inhibition of the p38 MAPK pathway partially abolished these effects. These findings are partly consistent with those of a previous study reporting an inverse association between p38 MAPK activity and alveolar bone destruction in a mouse model of periodontitis.^1^ In addition, Shikonin has been reported to activate ERK1 phosphorylation, and cyclic tensile stress promotes osteogenic differentiation of PDLSCs through the ERK1/2–ELK1 pathway.^20^, ^40^ Our results showed that the p‐ERK/ERK ratio increased under osteogenic induction, and this increase was further enhanced following Shikonin treatment. Therefore, Shikonin may also promote the osteogenic differentiation of PDLSCs through activation of the ERK1/2–ELK1 pathway. However, this possibility requires further experimental validation. Overall, our findings clarify the role of the p38 MAPK/ELK1 pathway in Shikonin‐induced upregulation of osteogenic differentiation in PDLSCs.

CEMP1 is a key regulator of cementogenesis and plays an essential role in periodontal tissue repair.^41^ During the inflammatory phase of periodontal healing, CEMP1 levels are reduced in the gingival crevicular fluid of patients with periodontitis.^42^ CEMP1 promotes the osteogenic differentiation of human gingival fibroblasts and supports periodontal regeneration.^22^, ^23^ In the present study, we also found that CEMP1 protein expression was increased during osteogenic differentiation of PDLSCs. In addition, we demonstrated that ELK1 binds to the *CEMP1* promoter and activates its transcription, thereby experimentally validating the JASPAR‐predicted binding sites. Furthermore, our findings indicate that ELK1 promotes the osteogenic differentiation of PDLSCs by directly transcriptionally activating the downstream target CEMP1. In hPDLSCs, crosstalk between CEMP1 and the MAPK/ERK pathway is partially consistent with the ELK1/CEMP1 interaction identified in our study.^43^ Notably, we also demonstrated that Shikonin promoted the osteogenic differentiation of PDLSCs, with activation of the involvement of the ELK1/CEMP1 axis contributing to this process. In summary, our findings further clarify the role of the ELK1/CEMP1 axis in regulating the osteogenic differentiation of PDLSCs.

Collectively, this study demonstrated that Shikonin enhanced the osteogenic differentiation of PDLSCs by modulating the p38 MAPK/ELK1 signaling axis, with the ELK1/CEMP1 axis contributing to this process. These findings support the potential of Shikonin as a therapeutic agent for promoting the osteogenic differentiation of PDLSCs. However, the lack of in vivo validation is a limitation of this study. Future studies will include in vivo experiments to verify the effects of Shikonin and evaluate its potential delivery strategies, including β‐1,3‐1,6‐glucan‐based formulations and local drug delivery systems. Overall, our findings suggest that Shikonin may serve as a potential therapeutic agent, and that the ELK1/CEMP1 axis may represent a promising molecular target for enhancing the osteogenic differentiation of PDLSCs.

## AUTHOR CONTRIBUTIONS

**Zeyu Wu**: Conceptualization; data curation; formal analysis; writing—original draft; writing—review and editing. **Chao Shan**: Methodology; project administration; writing—original draft; writing—review and editing. **Jie Song**: Methodology; software; writing—original draft; writing—review and editing. **Qian Yu**: Investigation; methodology; resources. **Bing‐jie Lian**: Supervision; validation; visualization. **Jin Zhao**: Funding acquisition; resources; software; supervision; writing—review and editing.

## CONFLICT OF INTEREST STATEMENT

The authors declare no conflicts of interest.

## ETHICS STATEMENT

PDLSCs were isolated from the periodontal ligaments of orthodontically extracted premolars from healthy donors with orthodontic reasons, under the approval of the Ethics Committee of The First Affiliated Hospital of Xinjiang Medical University hospital (Ethics Approval No. K202304‐19).

## Supporting information

Supporting Information S1

## Data Availability

The data that support the findings of this study are available from the corresponding author upon reasonable request.
