# Supplementary material for: Shikonin promotes osteogenic differentiation of periodontal ligament stem cells by modulating the p38 MAPK/ETS transcription factor 1/cementum protein 1 axis in vitro
Source: J Cell Commun Signal. 2026 Aug 8;20(3):e70104. doi: 10.1002/ccs3.70104 (PMC13452019; doi:10.1002/ccs3.70104)
Supplement: Supplementary file 1 [file CCS3-20-e70104-s001.pdf]

Supplementary Materials

Supplementary Figure and Figure legends

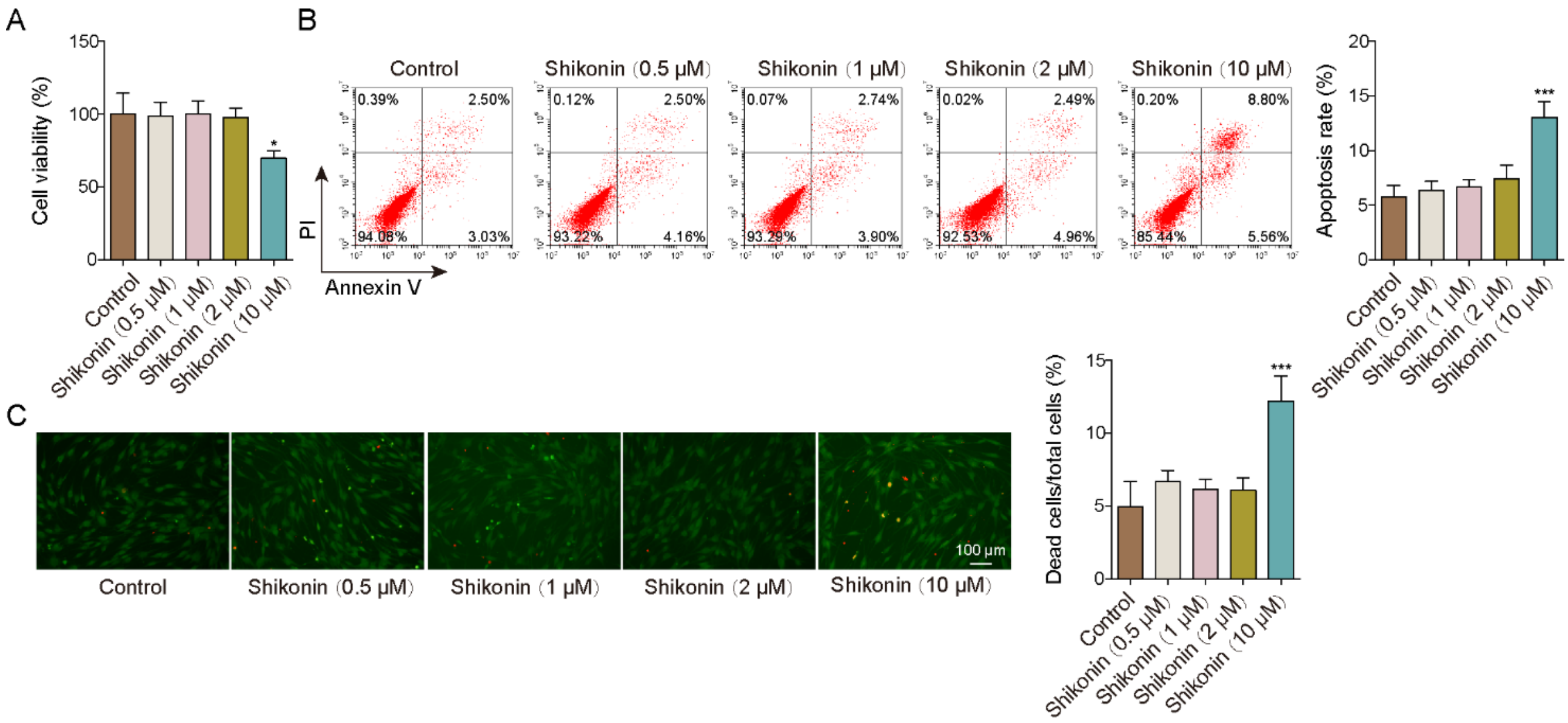

Supplementary Figure 1. Effect of Shikonin on the viability of PDLSCs.

PDLSCs were treated with Shikonin at concentrations of 0, 0.5, 1, 2, and 10 μM. (A) Cell viability was evaluated using the CCK-8 assay. (B) Flow cytometric analysis was performed to assess cell apoptosis. (C) A live/dead staining assay was performed to evaluate cell viability (scale bar = 100 μm). Tests for normality and homogeneity of variance were performed before statistical analysis. One-way ANOVA followed by Tukey's post hoc test was used for comparisons among multiple groups. Data are presented as the mean ± SD. Each experiment was independently performed at least thrice. N = 3 indicates three independent biological replicates, each consisting of three technical replicates. Statistical significance is indicated as follows: *$p < 0.05$, ***$p < 0.001$.

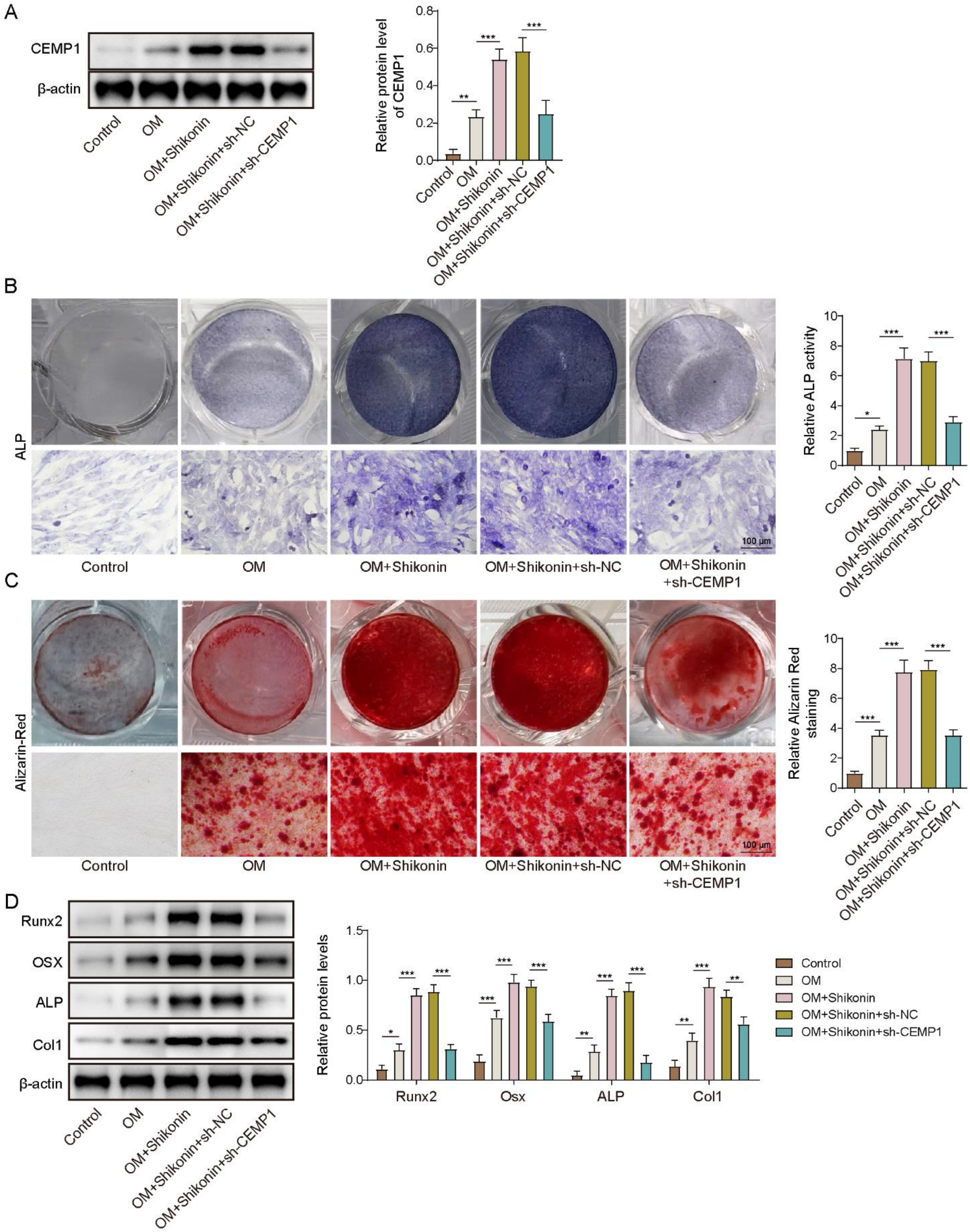

Supplementary Figure 2. Shikonin promoted osteogenic differentiation of PDLSCs by modulating CEMP1.

PDLSCs were transduced with the sh-CEMP1 lentiviral vector in the presence of Shikonin treatment

in OM. The cells were divided into the control, OM, OM + Shikonin, OM + Shikonin + sh-NC, and OM + Shikonin + sh-CEMP1 groups. (A) CEMP1 protein expression was determined by western blot analysis. (B) ALP staining was performed to evaluate ALP activity (scale bar = 100 μm). (C) Mineralized nodule formation was evaluated by ARS staining (scale bar = 100 μm). (D) The protein expression levels of the osteogenic markers Runx2, Osx, ALP, and Col1 were determined by western blot analysis. Tests for normality and homogeneity of variance were performed before statistical analysis. One-way ANOVA followed by Tukey's post hoc test was used for comparisons among multiple groups. Data are presented as the mean ± SD. Each experiment was independently performed at least thrice. N = 3 indicates three independent biological replicates, each consisting of three technical replicates. Statistical significance is indicated as follows: *$p < 0.05$, **$p < 0.01$, and ***$p < 0.001$.
